# Supplementary material for: Leaf Gas Exchange of Tomato Depends on Abscisic Acid and Jasmonic Acid in Response to Neighboring Plants under Different Soil Nitrogen Regimes
Source: Plants (Basel). 2020 Nov 29;9(12):1674. doi: 10.3390/plants9121674 (PMC7759899; doi:10.3390/plants9121674)
Supplement: Supplementary file 1 [file plants-09-01674-s001.pdf]

Supplementary Materials

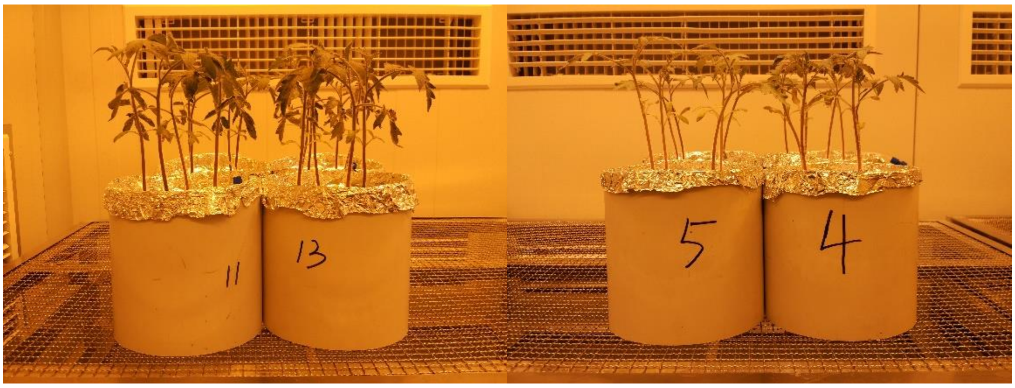

Figure S1. Morphological growth of competing plants under different N conditions at the end of the experiment. The robust plants on the left side were treated with N15 solution, while the puny plants on the right side were treated with N1 solution. N15 and N1 mean the levels of nitrogen in Hoagland solution were 15 mmol·L^{-1} and 1 mmol·L^{-1}, respectively.
